# Exhaled nitric oxide in a population-based study of Southern California Schoolchildren

**DOI:** 10.1186/1465-9921-10-28

**Published:** 2009-04-21

**Authors:** William S Linn, Edward B Rappaport, Kiros T Berhane, Tracy M Bastain, Edward L Avol, Frank D Gilliland

**Affiliations:** 1 Department of Preventive Medicine, Keck School of Medicine, University of Southern California, Los Angeles, California, USA; 2 Los Amigos Research & Education Institute, Downey, California, USA

## Abstract

**Background:**

Determinants of exhaled nitric oxide (FeNO) need to be understood better to maximize the value of FeNO measurement in clinical practice and research. Our aim was to identify significant predictors of FeNO in an initial cross-sectional survey of southern California schoolchildren, part of a larger longitudinal study of asthma incidence.

**Methods:**

During one school year, we measured FeNO at 100 ml/sec flow, using a validated offline technique, in 2568 children of age 7–10 yr. We estimated online (50 ml/sec flow) FeNO using a prediction equation from a separate smaller study with adjustment for offline measurement artifacts, and analyzed its relationship to clinical and demographic characteristics.

**Results:**

FeNO was lognormally distributed with geometric means ranging from 11 ppb in children without atopy or asthma to 16 ppb in children with allergic asthma. Although effects of atopy and asthma were highly significant, ranges of FeNO for children with and without those conditions overlapped substantially. FeNO was significantly higher in subjects aged > 9, compared to younger subjects. Asian-American boys showed significantly higher FeNO than children of all other sex/ethnic groups; Hispanics and African-Americans of both sexes averaged slightly higher than non-Hispanic whites. Increasing height-for-age had no significant effect, but increasing weight-for-height was associated with decreasing FeNO.

**Conclusion:**

FeNO measured offline is a useful biomarker for airway inflammation in large population-based studies. Further investigation of age, ethnicity, body-size, and genetic influences is needed, since they may contribute to substantial variation in FeNO.

## Introduction

The fractional concentration of exhaled nitric oxide (FeNO) is associated with the degree of airway inflammation and oxidative/nitrosative stress [1-5]. Measurement of FeNO has proven useful in clinical diagnosis and management of asthma [1-8] and in epidemiologic assessment of asthma and other respiratory diseases in children and adults [9-13]. We are conducting a longitudinal study of FeNO and its relationship to new-onset asthma in a large population-based sample of children participating in the Children's Health Study (CHS), an ongoing prospective cohort study of environmental and genetic determinants of respiratory health in southern California children [14-17]. We began annual FeNO measurements during the 2004–05 school year, testing more than 2500 children, most of whom were 8–9 years old and in grade 3, at schools in 13 communities representing a wide range of air quality. We used a validated offline technique [18-20] involving breath collection at schools with expiratory flow controlled near 100 ml/sec, refrigerated storage/transport of samples, and delayed analysis at a central laboratory. Using a calibration equation, the offline results were adjusted to equivalence with online FeNO measurements at the standard 50 ml/sec expiratory flow rate. This paper presents cross-sectional analyses of the initial year's data, intended to describe separate and interactive effects of allergy, asthma, and demographic factors on FeNO in this large, community-based population of children.

## Methods

### Study Population

Subjects were drawn from a CHS cohort of public-school students in 13 Southern California communities with different levels and types of air pollution, originally recruited in the 2002–3 school year when they were in kindergarten or grade 1. Informed assent was sought from each child, and informed consent from a parent or guardian, for FeNO testing. The protocol was reviewed and approved by the University of Southern California Institutional Review Board. Consent was obtained and testing was performed successfully on 2792 subjects out of 3544 available cohort members. As described in Additional file 1, some breath samples were rejected because of delayed analysis after an instrument failure, or anomalously high concentrations thought to result from outdoor light exposure; and a few subjects were excluded because of missing clinical or height/weight information. We attempted to test all participants without asthma and a sample of those with asthma. Thus, data were analyzable for 2568 subjects (92% of those tested, 72% of available cohort members). Table 1 presents descriptive data for this population.

**Table 1:** Selected Demographic Characteristics for 2568 Children with Valid FeNO measurements, Children's Health Study

	Girls (N = 1316)		Boys (N = 1252)	
Counts	Number	%	Number	%
Race/ethnicity				
Non-Hispanic White	452	34.4	440	35.1
Hispanic	731	55.6	688	55.0
African-American	27	2.0	22	1.8
Asian-American	40	3.0	40	3.2
Other/Unknown	66	5.0	62	4.9
Age				
< 8 yr	447	34.0	372	29.7
8–9 yr	644	48.9	592	47.3
>= 9 yr	225	17.1	288	23.0
Clinical Group				
Healthy	613	46.6	557	44.5
Allergy Only	608	46.2	562	44.9
Asthma Only	20	1.5	26	2.1
Allergy + Asthma	75	5.7	107	8.5
				
Summary Statistics	Mean ± SD	Range	Mean ± SD	Range

Age, yr	8.3 ± 0.6	6.4 – 10.4	8.4 ± 0.7	7.0 – 10.5
Height, cm	129 ± 7	108 – 157	131 ± 7	113 – 158
Weight, kg	30 ± 8	17 – 70	32 ± 8	19 – 72
Residual height-for-age, cm^[a]^	0 ± 6	-18 – +24	0 ± 6	-17 – +22
Residual weight-for-height, kg^[b]^	0 ± 5	-21 – +28	0 ± 6	-17 – +35

^[a] ^Observed height minus predicted height for subject's age; predicted values determined from cubic polynomial fit to data for all subjects of the same sex in this population (see text).

^[b] ^Observed weight minus predicted weight for subject's height; predicted values determined as described in note [a].

### Subject Characterization

Questionnaires, distributed to children at school and completed by parents, were used to determine children's demographic and allergy/asthma characteristics [14,16]. Using questionnaire responses, each child was assigned to one of the following clinical groups for statistical comparison: healthy (no report of asthma or allergy in questionnaires from 2002 through 2004), respiratory allergy without asthma, asthma without respiratory allergy, or asthma with respiratory allergy. (Children with reports of wheeze but no asthma or allergy, initially considered as a separate group, were ultimately included in the healthy group because their FeNO showed nonsignificant differences from other healthy subjects.) Children were classified as having asthma based on parents' report of a physician diagnosis of asthma. In an earlier validation study on a subsample, 95% of such reports were corroborated by medical record review [21]. In analyses to explore effects of asthma severity and medication usage, children with evidence of asthma were assigned to one of the following groups: 1) reported asthma medication use without an asthma diagnosis, 2) physician diagnosis of asthma but no medication use, 3) active asthma with rescue medication only (i.e. as-needed bronchodilators), 4) active asthma with controller medication (i.e. regular long-acting bronchodilators, corticosteroids, or leukotriene inhibitors). Subjects were also classified according to their age on the day of testing. Because a preliminary analysis indicated that FeNO variation was not linear with age, age strata were defined with cutpoints at 8.00 and 9.00 yr. (An alternative analysis by quartiles of age gave similar results.) Height and weight were measured on the day of testing and were compared with growth charts from the Centers for Disease Control (CDC) [22]. Weight-for-height distribution was substantially above CDC norms. Therefore, we calculated predictions of weight-for-height and height-for-age for our population, emulating the CDC approach. We used least-squares regression to determine cubic polynomial prediction equations for each variable, separately for each sex but pooling all ethnic groups. We then calculated residual (observed minus predicted) height-for-age and weight-for-height for each subject, and used these residuals as predictor variables in statistical modeling of FeNO. Residuals (summary statistics in Table 1) correlated weakly with each other or with age (r between -0.1 and +0.1), unlike height and weight per se (r between +0.3 and +0.7), thus allowing more stable model estimates of body size effects on FeNO independent of age.

### Sample Collection, Transport, and Analysis

Exhaled breath collection was performed at schools, usually from midmorning to noon to avoid traffic-related peaks of ambient NO and possible effects of recent eating on FeNO. Each community was visited at least twice in different seasons, to minimize confounding of location and season effects. Health status at testing was evaluated by a brief questionnaire; subjects with symptoms of acute respiratory infection within the past 3 days were excluded or rescheduled. The methodology for offline measurement of FeNO was validated by a small-scale a priori comparison against conventional online measurements [18], and by a larger-scale post hoc comparison study [19,20] described later. Breath was collected in aluminized Mylar bags using commercial breath sampling kits (Sievers Division, GE Analytical Instruments, Boulder, Colorado, USA), following the manufacturer's instructions based on American Thoracic Society (ATS) recommendations [1]. The target expiratory flow and pressure were 100 ml/sec and 19 cmH_2_O respectively. The subject took 2–3 initial deep breaths through the kit's NO scrubber – which was less than 100% effective [19,20] – followed by a near-vital-capacity breath and immediate controlled exhalation through the sampling kit. Deadspace air was discarded for approximately 3 sec, then 0.5–1 L of exhalate was collected in the bag. Bag samples of ambient air, collected similarly through the breath sampling kit by removing the NO scrubber and drawing the sample with a syringe, were used to estimate each subject's ambient NO exposure at the time of testing. All bag samples were stored in coolers and transported to a central laboratory, where NO was measured using a standard chemiluminescent analyzer system (Sievers Division). The resulting offline FeNO data were converted to estimates of "standard" FeNO as would be measured online at 50 ml/sec expiratory flow [3], using a statistical model determined in the post hoc comparison study [19,20], which involved 362 children (including 309 from this population) measured on and off line in the same testing session. The statistical model, described in Additional file 1, accounted for artifacts in offline measurements due to varying ambient NO concentrations and varying lag times between breath collection and analysis.

Commercial software (SAS Institute, Cary, NC) was used for data quality checking and subsequent analysis. After exclusion of samples that developed problems after collection and subjects with incomplete demographic or clinical information (see Additional file 1), 2,568 subjects had usable data. Predicted online FeNO (50 ml/sec) was tested for effects of asthma status and demographic characteristics (sex, ethnic group, age, community of residence) using regression models fit with SAS procedure MIXED. Previously mentioned grouping variables and community of residence were treated as fixed factors. Initially, main effects of each factor were tested; subsequently, two-way interactions between grouping factors were tested one at a time, by adding the interaction term to the original main-effects model. To verify that significant differences were not artifacts of the process used to predict online FeNO, the same analytical model was applied to raw offline FeNO measurements (originally measured values, not adjusted for any technical artifacts). Analyses were performed on log-transformed FeNO data to obtain more nearly normal distributions. Effects with 2-sided P < 0.05 were considered statistically significant.

## Results

### Overview

**Figure 1:**
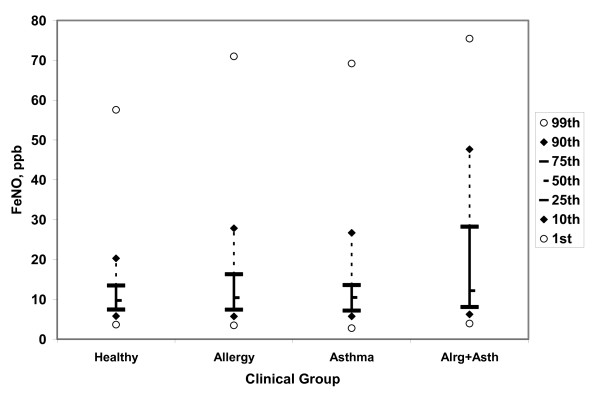
**Percentile distributions of estimated online FeNO for healthy, allergy-only, asthma-only, and allergy-plus-asthma subjects**. See Table 1 for number of subjects in each group. Allergy-only and allergy-plus-asthma groups are significantly (P < 0.0001) different from healthy group; asthma-only group is not significantly different.

Table 1 provides a statistical description of the 2,568 subjects with analyzable data. They generally resembled the entire tested population of 2,792, although nonwhite and Hispanic subjects were somewhat underrepresented (see Additional file 1). Table 2 shows key results from regression analysis of predicted conventional online FeNO. Additional file 1 provides more detailed results. Table 3 shows key results from the analysis of raw offline FeNO data. Proportional changes between groups were similar, and the pattern of significant group differences was identical, with raw offline and predicted online data. Thus, group differences reflect underlying biology and are not artifacts of the adjustment or estimation process. FeNO varied appreciably by ethnicity, age group, asthma status, and respiratory allergy status. Figure 1 illustrates the distribution of predicted online FeNO by clinical group. Values diverged (mostly in the upper halves of the distributions) but still overlapped considerably: the 75^th ^percentile for allergic-asthmatic subjects was similar to the 90th percentiles for subjects with allergy only or asthma only, and the 95th percentile for healthy subjects. Receiver operating characteristic (ROC) curves (not shown) were examined to determine how well the FeNO measurement could discriminate individuals with allergy or asthma from those without these conditions. To minimize confounding by nonclinical factors, ROC analyses were limited to non-Hispanic white and Hispanic subjects, and performed separately for subjects below and above age 9. Areas under ROC curves were 0.60 or less for allergy versus no allergy, or for any asthma versus no asthma. (Area of 0.50 would indicate no ability to detect allergy or asthma, and 1.0 would indicate correct classification of every subject.) The ability to classify active asthma with recurrent clinically significant wheezing, or with medication use in the past year, was slightly better, with areas under ROC curves of 0.66 to 0.68.

**Table 2:** Geometric Mean FeNO* among Children by Demographic and Clinical Determinants of FeNO, CHS 2003–4 Subjects with Valid FeNO Data**

GROUP	Geometric Mean FeNO (ppb)	95% Confidence Interval of Geometric Mean	P Value for Group Main Effect
Female	12.63	(11.75, 13.56)	0.69
Male (reference)	12.75	(11.88, 13.68)	
			
Non-Hispanic White (reference)	11.56	(10.85, 12.31)	<0.0001
Hispanic‡	12.54	(11.84, 13.29)	
African American	12.65	(10.58, 15.13)	
Asian American‡	16.10	(14.00, 18.53)	
Other	11.12	(9.93, 12.45)	
			
Age < 8‡	12.15	(11.29, 13.08)	<0.0001
Age 8–9‡	12.25	(11.42, 13.13)	
Age >= 9 (reference)	13.72	(12.62, 14.91)	
			
Healthy (reference)	10.95	(10.34, 11.61)	<0.0001
Allergy only‡	12.39	(11.71, 13.10)	
Asthma only	12.07	(10.09, 14.45)	
Allergy + Asthma‡	15.81	(14.33, 17.43)	

* Regression Predicted Online FeNO (50 ml/sec).

** Analytical model includes main effects of sex, ethnic group, clinical group, age stratum, height-for-age, weight-for-height, and community; see Additional file 1 for additional results.

‡Different from reference group, P < 0.001.

**Table 3:** Regression Results: Unadjusted Offline FeNO (100 ml/sec) by Demographic and Clinical Category, CHS 2003–4 Subjects with Valid FeNO Data*

GROUP	Geometric Mean FeNO	95% Confidence Interval of Geometric Mean	P Value for Group Main Effect
Female	8.31	(7.85, 8.80)	0.62
Male (reference)	8.39	(7.93, 8.87)	
			
Non-Hispanic White (reference)	7.85	(7.47, 8.25)	<0.0001
Hispanic‡	8.29	(7.92, 8.68)	
African American	8.31	(7.21, 9.57)	
Asian American‡	9.98	(8.93, 11.15)	
Other	7.52	(6.88, 8.22)	
			
Age < 8‡	8.17	(7.71, 8.66)	<0.0001
Age 8–9‡	8.13	(7.69, 8.59)	
Age >= 9 (reference)	8.77	(8.21, 9.36)	
			
Healthy (reference)	7.50	(7.16, 7.85)	<0.0001
Allergy only‡	8.40	(8.03, 8.78)	
Asthma only	7.83	(6.79, 9.02)	
Allergy + Asthma‡	9.86	(9.13, 10.65)	

*Analytical model includes main effects of sex, ethnic group, clinical group, age stratum, height-for-age, weight-for-height, and community.

‡Different from reference group, P < 0.001.

### Variation in FeNO by Clinical Status

Asthma/allergy groups showed highly significant (P < 0.0001) overall differences in FeNO. Asthma-without-allergy and allergy-without-asthma groups showed roughly similar increments relative to healthy subjects, while children with both respiratory allergy and asthma showed substantially larger increments. The asthma-without-allergy group, considered by itself, was not significantly different from healthy subjects, possibly because its small size limited statistical power of the group-comparison test. In a model including separate and interactive effects of allergy and asthma, both main effects were significant (P < 0.001) but the interaction did not reach statistical significance. Again, the interaction test had low power due to the small number of non-allergic asthmatics. Other 2-way interactions of grouping factors were nonsignificant, with the exception of sex and ethnicity, discussed below.

To investigate the relationship of asthma activity and medication use to FeNO, we examined geometric mean FeNO for subgroups of physician-diagnosed asthmatic or asthma-medication-using subjects, adjusted for nonclinical factors as above. Detailed results are given in Additional file 1. Overall differences by medication status were statistically significant (P < 0.05) but small: geometric means were approximately 12 ppb in diagnosed asthmatics with no medication use and in medication users with no asthma diagnosis, 13 in diagnosed asthmatics using rescue medication only, and 17 in those using rescue plus controller medications. Thus, in our population more clinically persistent asthma was associated with a higher FeNO, which was not entirely counteracted by more intensive medication use.

### Demographic Determinants of FeNO

**Figure 2:**
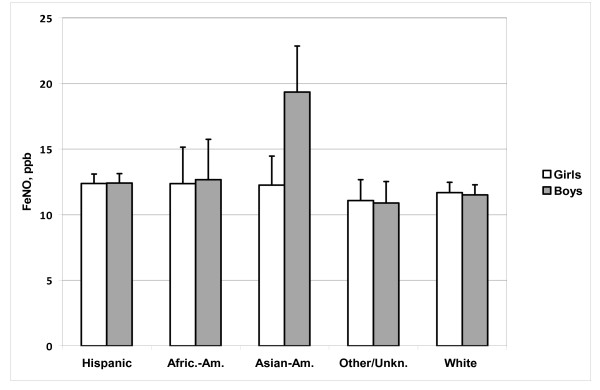
**Estimated online FeNO by sex and ethnic group, adjusted for clinical status, age, body size, and community effects**. Bar indicates geometric mean, flag indicates upper 95% confidence limit of geometric mean. See Table 1 for number of subjects in each group. Significant differences: Hispanic > white, P = 0.02; Asian > white, P < 0.0001; Asian boys > Asian girls, P < 0.0001.

Age and ethnic group were significantly associated with FeNO (P < 0.0001 for either main effect). Point estimates for both younger age strata were about 11% lower than that for the 9 year-and-older stratum, after adjustment for other factors. Compared to the non-Hispanic white reference group, Asian-Americans showed a relatively large significant increase. Hispanics and African-Americans showed smaller increases, significant in Hispanics but not significant in African-Americans, probably because their smaller numbers limited statistical power. Elevated FeNO in Asian-Americans occurred primarily in boys. Figure 2 summarizes results from an analytical model including an interactive effect of ethnic group and sex, which was significant (P < 0.01), with a markedly larger estimated increment in Asian-American boys than girls (point estimates 81% and 7% respectively, relative to non-Hispanic whites of the same sex). The increment was observable in healthy and in allergic boys; asthmatics were too few for reliable comparison.

### Effect of Body Size on FeNO

Weight was associated with FeNO levels, while height was not, after accounting for effects of age. Residual weight-for-height (weight difference from a typical subject of the same sex and height, without regard to age) showed a small statistically significant (P < 0.01) negative effect, estimated at 0.7% decrease in FeNO per kilogram. For example, in healthy >9-year-old non-Hispanic white boys of typical height, an increase of 20 kg above typical weight would predict a decrease in FeNO from 12.5 to 10.8 ppb. An alternative analysis by quartiles of residual weight-for-height (data not shown) indicated that this effect was present over most or all of the weight range, i.e. not driven by a minority of overweight or underweight individuals. Although weight-for-height was significantly higher in Hispanics than in Non-Hispanic whites, the weight effect did not explain those groups' significant difference in FeNO (see Additional file 1). Too few data were available to compare other ethnic groups in the same manner.

## Discussion

### Overview

This large population-based study evaluated FeNO in a community population of schoolchildren in a region with generally milder climate, but generally more severe air pollution, than locations of other FeNO surveys. The results corroborated previous findings that respiratory allergy, asthma, Asian ancestry, and effects of maturation tend to increase children's FeNO. New findings included slight but significant elevation of FeNO in Hispanic relative to non-Hispanic white children, and a slight but significant tendency for FeNO to decrease with increasing weight in children of a given height.

### Effect of Clinical Status

We found highly significant positive effects of respiratory allergy or asthma on FeNO. Increases due to allergy and/or asthma have been found in numerous earlier smaller-scale studies of children and adults [4-10,23-26]. Levels of FeNO were, however, not greatly elevated in our sample of asthma cases in comparison with our healthy subjects. The relatively small differences (compared to differences found in many clinic-based studies) may reflect mildness of asthma in this population-based sample, relatively effective suppression of elevated NO by asthma medication, or misclassification of asthma status (based only on parents' questionnaire reports). Although most questionnaire reports should be valid, as judged by medical record review in a subsample [21], "false positive" and "false negative" asthma classifications still might be more likely here than in a clinic-based study with uniform diagnostic testing. If so, the FeNO difference between subjects with and without asthma would be underestimated here. The same would be true concerning allergy, even less clearly definable than asthma. Subject to these limitations, the allergy effect appeared to be equal or larger, and more significant, than the asthma effect in this population. Point estimates suggested that the combined effect of allergy and asthma was more than additive, but statistical tests did not confirm synergism. A previous population-based survey of Canadian children mostly older than our subjects, employing online FeNO testing and health questionnaires, found that mean FeNO varied by health status in the same manner as we observed, i.e. allergy + asthma > allergy only > asthma only > healthy [23]. In a smaller population-based study of Finnish children, with a wider age range than ours and with individual medical testing to establish health status, atopy was a significant predictor of increased FeNO, while asthma was not [26]. Thus, these geographically and methodologically diverse population-based studies suggest that allergy increases FeNO as does asthma.

Typical FeNO levels in our population were slightly lower than in the Canadian study (consistent with its older age range), but slightly higher than in subjects of similar age in the Finnish study. Some difference in FeNO readings between studies may be expected, given inherent variability of NO analyzers [27,28] and standard gases, as well as differences in subject selection, clinical classification, and methods of calculating summary statistics.

A potentially important finding, which is not new but seems to have received little attention, is that an appreciable percentage of clinically healthy subjects have FeNO levels in the range usually associated with poorly controlled asthma. For our Hispanic and non-Hispanic white subjects reported to be free from allergy or asthma, the 95th and 99th percentiles of FeNO were near 30 and 60 ppb respectively, and the highest values were near 100 ppb. We cannot entirely rule out the possibility that some of these high values resulted from artifacts, similar to the known incidents described in Additional file 1. Even so, our overall results in healthy subjects are consistent with those found by Kovesi et al. [24] using state-of-the-art online testing equipment. In their survey, about 2% of healthy non-Hispanic white subjects aged 9–12 exceeded 50 ppb ([24] Figure 1). Further investigation is warranted to determine whether these atypically high concentrations reflect unacknowledged/undiagnosed respiratory disease, nonrespiratory pathology resulting in increased NO excretion through the respiratory tract, effects of air pollutants, clinically benign genetic variants, or some other cause. As clinical phenotypes are defined more fully in this ongoing study, their associations with FeNO will be re-examined.

### Effects of Growth

Previous studies of children have agreed that FeNO increases with growth, but have disagreed concerning the relative importance of age, height, and weight or body mass index to this increase. These apparent inconsistencies may reflect different age ranges and different clinical exclusion criteria in different studies, as well as different strategies for statistical modeling with multiple positively correlated predictor variables. Our cross-sectional analysis of a population with a narrow age range has limited ability to detect growth effects. Nevertheless, we have shown a highly significant age-related increase beginning around age 9, in general agreement with previous studies in North America and Europe [23-26]. We found only a slight, nonsignificant tendency to higher FeNO in taller versus shorter subjects of the same age, but found a significant tendency to lower FeNO in heavier-weight versus lighter-weight subjects of a given height. This effect of weight (or body mass index), if confirmed in other populations, may have mechanistic implications, but seems too small to impact the interpretation of clinical test results. Clearly, however, changes in FeNO due to maturation must be accounted for in some manner when interpreting FeNO measurements in children. Our results suggest age as the most appropriate adjustment factor, but they may not be generalizable to younger or older children.

### Ethnic Differences

We found higher adjusted mean FeNO in Hispanic children than in non-Hispanic white children. To our knowledge, this ethnic difference has not been reported previously. Our observation of elevated FeNO in Asian-descended boys, relative to European-descended boys and Asian-descended girls, is consistent with previous findings in Canada [24] and Hong Kong [29]. A genetic basis for this phenomenon is possible, given that an environmental influence specific to Asian-descended boys, acting consistently in all these locations, seems unlikely. Our results do not entirely conform with the earlier findings, in that we found little FeNO increase in Asian-American relative to non-Hispanic white girls. Further studies with large samples from both ethnic groups are needed to clarify the gender-ethnicity interaction. Unfortunately, we did not have a large enough sample of African-American children to adequately examine difference from other groups. The same was true in the Canadian study [23]. However, both studies suggest that African-descended children fall below Asian- and above European-descended children in their range of FeNO. Similarly, in an earlier clinic-based multinational study of healthy children [25], non-Caucasian (predominantly African-American) children showed a borderline-significant increase in FeNO relative to Caucasian children.

### Validation of Methodology

We used an offline technique for measurement of FeNO. We have reported elsewhere a strong correlation of offline and online FeNO measurements in our study and have developed a calibration equation to convert offline measurement at 100 ml/s to the equivalent of online FeNO measured at 50 ml/s [19,20]. We found a positive effect of concurrent ambient NO concentration on FeNO that could reflect contamination of collected breath samples by ambient NO, and/or an acute biological response (e.g. airway inflammation) to NO-containing ambient pollution, which might be expected on the basis of previous findings in asthma panel studies [30,31]. From the on-versus-offline comparison data, we concluded that the association of offline FeNO with ambient NO in our surveys is largely the result of a contamination artifact [19,20]. Whatever the mechanism(s), short-term variations in ambient NO clearly can influence FeNO measurements, and should be taken into account in analysis of FeNO changes. Time-dependent changes in NO concentration of stored breath samples may create additional artifacts that should be accounted for, and samples must be protected from exposure to outdoor light (and presumably other strong light) during storage. With these caveats, offline FeNO measurement with delayed analysis appears to be a valid and practical tool for large-scale respiratory health surveys. Even so, direct online FeNO measurement is preferable, as it allows immediate and objective documentation of test quality. Reasonably portable online NO analyzers are now available and have been used successfully in large-scale surveys [23,24]. Although new devices are available, delayed offline measurement may be more practical in some circumstances. Even with careful online testing, comparability of FeNO measurements across different studies is not assured, since different manufacturers' analyzers may read breath NO differently even when calibrated with the same standard gases [27,28]. This suggests that other trace breath constituents may enhance or suppress NO chemiluminescence, and act differently with different reaction chamber designs. Whatever their cause, these differences dictate caution in inter-study data comparisons.

## Conclusion

This study has demonstrated the feasibility of large-scale field surveys of FeNO using offline breath collection and delayed laboratory analysis. In our population-based study, children's FeNO varied by respiratory allergy, asthma, ethnic origin (particularly in boys of Asian descent), and increasing age, as has been previously reported. An apparently new finding of elevated FeNO in Hispanic children relative to non-Hispanic white children warrants further investigation. The results reemphasize that FeNO may depend importantly on host factors other than respiratory health. These other sources of variation may complicate the interpretation of clinical FeNO tests.

## Competing interests

The authors declare that they have no competing interests.

## Authors' contributions

WL wrote the manuscript, performed statistical analyses, and oversaw field and laboratory work. ER performed data management, statistical analyses, and manuscript review. KB provided statistical review and oversight, and manuscript review. TB and EA provided project scientific oversight and manuscript review. FG had primary responsibility for scientific design, scientific oversight of this and related projects, and manuscript review.

## Supplementary Material

Additional file 1 **Additional Details of Methodology and Results**. Gives details of offline FeNO prediction model, excluded data, weight effects on measured FeNO, medication effects on measured FeNO. Click here for file
