# Supplementary material for: Exhaled nitric oxide in a population-based study of Southern California Schoolchildren
Source: Respir Res. 2009 Apr 21;10(1):28. doi: 10.1186/1465-9921-10-28 (PMC2678086; doi:10.1186/1465-9921-10-28)
Supplement: Additional file 1 — Additional Details of Methodology and Results. Gives details of offline FeNO prediction model, excluded data, weight effects on measured FeNO, medication effects on measured FeNO. [file 1465-9921-10-28-S1.doc]

**ADDITIONAL DETAILS OF METHODOLOGY AND RESULTS**

**Prediction of FeNO at 50 ml/sec Exhalation from Measurement Offline at 100 ml/sec Exhalation**

From data on 362 subjects (309 from the population reported in this paper plus 53 additional similar individuals), the best prediction model found (R^2^ = 0.94) was of the form

[predicted online FeNO] = [measured offline FeNO]*[proportionality factor],

with the proportionality factor depending on the level of offline FeNO and on concurrent ambient NO concentration, an important artifactual influence. Concurrent ambient NO was expressed as a proportion of offline FeNO, and different regression slopes were estimated for proportions above and below 1, because of restriction of range below 1. Other possible artifacts due to sample bag aging, residual bag contamination, and deviations from prescribed expiratory flow rate (as judged by the test technician) were negligible, as determined in preliminary analyses. However, the artifactual influence of lag time between breath collection and NO analysis was found to be significant. It was accounted for by estimating separate equations for samples analyzed on the day of collection, and samples held overnight before analysis. Within those categories, variation in lag time did not have a significant influence.

Prediction equations were

P = M*[exp(0.182 + 0.141*log(M) – 0.139*log(max(1, A/M)) – 0.0360*log(min(1, A/M)))]

for same-day samples; and

P = M*[exp(0.103 + 0.158*log(M) – 0.278*log(max(1, A/M)) – 0.0439*log(min(1, A/M)))]

for overnight samples. In these equations P represents predicted online FeNO at 50 ml/sec, M represents offline FeNO measured at 100 ml/sec, max() indicates the larger quantity in parentheses, min() indicates the smaller quantity in parentheses, and A represents estimated ambient NO at the time of offline collection.

**Samples Excluded from Data Analyses**

A total of 2792 subjects provided bag samples of breath that were considered valid at the time of collection. In the initial data review, batches of samples from two test sessions conducted entirely outdoors were found to have an improbably large proportion of high NO concentrations. That anomaly was attributed to artifacts from photochemical reactions due to direct exposure to outdoor light that penetrated the Mylar bags. Positive artifacts were demonstrated in a subsequent experiment comparing paired bag samples, with one of each pair exposed to outdoor light and one kept indoors. The 31 affected subjects (1.1%) were excluded from data analyses. Later, a preliminary review of longitudinal data (unpublished) revealed a few outlying high FeNO values in the initial year, prompting a case-by-case review of field technicians’ notes, which identified a further 124 subjects (4.4%) whose samples may have been exposed to outdoor light. Their NO concentrations were significantly (P < 0.01) elevated compared to other subjects', so they were also excluded from analysis. A further 51 subjects (1.8%) were excluded from data analyses because an NO analyzer failure necessitated delayed measurement of their samples (lag time up to 54 hr) with different equipment, introducing extra potential sources of bias. A further 18 subjects (0.6%) with valid FeNO data were excluded because of missing predictor data – height, weight, or clinical status. Chi-square tests were performed to compare excluded and included subjects. There were no significant differences by sex, age category, or clinical category (ignoring the unclassifiable category). There was a significant difference by ethnicity: proportions of Hispanic, African-American, and Asian-American subjects tended to be higher in excluded subjects.

**Results from "Comprehensive" Main-Effects Model**

Table S-1 presents model results as a point estimate of FeNO for reference subjects (healthy Non-Hispanic white males aged >=9, with height-for-age and weight-for-height 100% of predicted), and an estimated percentage change in FeNO due to each factor in the model.

**Additional Results Concerning Weight Effects on FeNO**

Residual weight-for-height varied by clinical status and ethnic group, being significantly elevated in allergic asthmatics (mean +1.1 kg, P = 0.008) and in Hispanic subjects (+1.5 kg, P < 0.0001), but decreased in Non-Hispanic white subjects (-0.6 kg, P = 0.02). As Figure 3 in the main paper shows, Hispanics averaged similar or slightly shorter in height, but appreciably heavier as indicated by body mass index, in comparison with non-Hispanic whites of the same age range. (Other ethnic groups had too few subjects for reliable comparison.) Adjustment for weight difference increased the estimate of FeNO difference between Hispanics and non-Hispanic whites. In a model not including body size effects, the estimated increment in Hispanics was 7.5%, still significant. Additional analyses were performed on Non-Hispanic whites and Hispanics separately, employing new cubic polynomial predictions of weight-for-height specific to each ethnic group. Resulting estimates of residual-weight-for-height effect were -0.74%/kg (P = 0.006) for Hispanics, and -0.56%/kg (P = 0.19) for Non-Hispanic whites. Thus, the negative effect of weight was more obvious in Hispanics, but probably not unique to them.

**Additional Results Concerning Asthma Medications' Effects on FeNO**

To investigate the relationship of asthma activity and medication use to FeNO, we examined geometric mean FeNO for subgroups of physician-diagnosed asthmatic or asthma-medication-using subjects, adjusted for nonclinical factors as above. Table S-2 shows results. In a model that tested for main and interactive effects of allergy and medication status, none of the subgroup differences were statistically significant. However, with allergic and non-allergic subjects pooled, differences by medication status were statistically significant (P = 0.02), showing a positive trend from no medication to rescue medication to controller medication. Thus, more clinically persistent asthma was associated with an increase in FeNO, which was not entirely counteracted by more intensive medication use. There was no way to verify that medication reported on questionnaires was actually being used at the time of FeNO testing; thus, some misclassification of actual medication status may have occurred, leading to underestimation of differences between categories.

Table S-1. Adjusted relative differences of FeNO for demographic and clinical groups, CHS 2003-4, based on "comprehensive" main-effects model^[a]^

| EFFECT | ESTIMATE (95% conf. limits) | P value^[c]^ |
| --- | --- | --- |
| Reference estimate^[b]^ | 12.6 ppb (11.3, 14.0) |  |
| Female | -0.9% (-5.5, +3.8) | 0.69 |
| Hispanic | +8.5% (+2.5, +14.9) | 0.004 |
| African-American | +9.5% (-8.5, +31.0) | 0.31 |
| Asian-American | +39.3% (+21.1, +60.3) | <0.0001 |
| Other/unknown ethnicity | -3.8% (-14.0, +7.7) | 0.49 |
| Allergy, no asthma | +13.0% (+7.7, +18.8) | <0.0001 |
| Asthma, no allergy | +10.2% (-7.7, +31.6) | 0.27 |
| Allergy + asthma | +44.3% (+31.2, +58.7) | <0.0001 |
| Age < 8 yr | -11.4% (-17.2, -5.2) | 0.0004 |
| Age between 8 and 9 yr | -10.7% (-16.1, -4.9) | 0.0003 |
| Increased height-for-age^[d]^ | +0.30%/cm (-0.12, +0.71) | 0.15 |
| Increased weight-for-height^[d]^ | -0.74%/kg (-1.17, -0.32) | 0.0005 |

^[a]^ Overall differences between communities were significant at P < 0.0001; individual community estimates not shown.

^[b]^ For healthy non-Hispanic white male subjects aged 9 years or older, with normal height-for-age and weight-for-height (residual = 0) based on prediction equations estimated for this population (see Methods section of main paper).

^[c]^ For comparison against reference sex, ethnic, clinical, or age group (see note [b]); or against zero slope.

^[d]^ Based on norms estimated for this population (see Methods section of main paper).

Table S-2. Adjusted Geometric Mean FeNO (95% Confidence Limits) in 307 Subjects with Physician-Diagnosed Asthma and/or Asthma Medication Use in Past Year*, CHS 2003-4

|  | Not Allergic | Allergic | All Subjects |
| --- | --- | --- | --- |
| Medication, no asthma diagnosis | 11.8 (8.3, 16.7)  N = 24 | 11.8 (9.0, 15.3)  N = 55 | 11.8 (9.4, 15.0)  N = 79 |
| Asthma diagnosis, no medication | 10.6 (7.6, 14.8)  N = 26 | 12.2 (9.5, 15.6)  N = 66 | 11.8 (9.4, 14.7)  N = 92 |
| Asthma diagnosis, rescue medication only | 9.9 (6.3, 15.5)  N = 13 | 14.2 (11.1, 18.1)  N = 61 | 13.3 (10.5, 16.9)  N = 74 |
| Asthma diagnosis, controller medication | 12.7 (7.0, 23.0)  N = 7 | 17.5 (13.6, 22.6)  N = 55 | 17.0 (13.3, 21.7)  N = 62 |

*When broken down by both allergy and asthma-medication status (left-hand and center data columns), means are not significantly different (P for allergy status = 0.07, P for asthma medication status or interaction > 0.4). With all medication-using subjects pooled, ignoring allergy status, means by asthma medication status (right-hand column) are significantly different (P = 0.02). These results exclude 2 subjects with reported asthma medication but no information on specific drugs used.
